# Immune responses in rodent whole eye transplantation: elucidation and preliminary investigations into rejection diagnosis and monitoring

**DOI:** 10.3389/fimmu.2025.1475055

**Published:** 2025-01-29

**Authors:** Bing Li, Yong Wang, Charles R. Owens, Touka Banaee, Charleen T. Chu, Kayvon Jabbari, Anna D. Lee, Neil J. Khatter, Alan G. Palestine, An-Jey A. Su, Christene A. Huang, Kia M. Washington

**Affiliations:** ^1^ Division of Plastic Surgery, Department of Surgery, University of Colorado Anschutz Medical Campus, Aurora, CO, United States; ^2^ Department of Ophthalmology and Visual Sciences, University of Texas Medical Branch, Galveston, TX, United States; ^3^ Department of Pathology, University of Pittsburgh School of Medicine, Pittsburgh, PA, United States; ^4^ Sue Anschutz-Rogers Eye Center, Department of Ophthalmology, University of Colorado Anschutz Medical Campus, Aurora, CO, United States

**Keywords:** whole eye transplantation, vascularized composite allotransplantation, immune rejection, diagnostic strategies, monitoring techniques, non-invasive biomarkers

## Abstract

**Background:**

Whole Eye Transplantation (WET) offers potential for vision restoration but is hindered by the complex challenge of immune rejection. Understanding and closely monitoring these immunological responses is crucial for advancing WET. This study delves into the timeline and nature of immune responses in a rodent model of WET without immunosuppression, aiming to elucidate a detailed picture of the immune landscape post-transplantation and establish innovative diagnostic and monitoring methods.

**Methods:**

We employed a multi-faceted approach to analyze immune responses post-WET, including assessments of gross changes in corneal transparency, thickness, and skin condition. Histopathological examinations of both ocular and surrounding skin tissues provided insights into cellular changes, complemented by ocular RT-qPCR for molecular analysis. Serological analysis was employed to quantify cytokines, chemokines, and donor-specific antibodies, aiming to identify potential biomarkers correlating with WET rejection and to validate the presence of antibody-mediated rejection. These methodologies collectively contribute to the development of non-invasive diagnostic and monitoring strategies for WET.

**Results:**

Our study revealed a rapid and acute immune response following WET, characterized by an early innate immune response dominated by complement involvement, and infiltration of neutrophils and monocytes by post-operative day (POD) 2. This was succeeded by an acute T-cell-mediated immune reaction, predominantly involving T helper 1 (Th1) cells and cytotoxic T lymphocytes (CTLs). The presence of donor specific antibody (DSA) and indications of pyroptosis in the early phases of rejection were observed. Notably, the early elevation of serum CXCL10 by POD4, coupled with ocular CD3+ cell infiltration, emerged as a potential early biomarker for WET rejection. Additionally, corneal transparency grading proved effective as a non-invasive monitoring tool.

**Conclusion:**

This study offers a first-time comprehensive exploration of immune responses in WET, unveiling rapid and complex rejection mechanisms. The identification of early biomarkers and the development of non-invasive monitoring techniques significantly advance our understanding of WET rejection. Additionally, these findings establish an essential baseline for future research in this evolving field.

## 1 Introduction

Blindness is a global public health issue that can severely debilitate individuals, their families and the society as a whole. The World Health Organization estimated that 43.3 million people were blind in 2020. Furthermore, an additional 20 million individuals are predicted to be diagnosed with blindness over the next 30 years, with the total blind population projected to exceed 61 million by 2050 (1). Optic nerve regeneration technology, such as stem cell therapy, gene therapy, and tissue engineering, has shown promise in restoring vision (2–8). Unfortunately, these techniques often prove insufficient to address all of the underlying pathologies that lead to vision loss, such as traumatic injury (9) or vasculopathy and retinopathy caused by systemic diseases like diabetes (10, 11). In such cases, whole eye transplantation (WET) represents a promising approach with the potential to contribute to future strategies for addressing untreatable vision loss.

WET, a specialized form of vascularized composite allotransplantation (VCA), entails transplanting not only the entire eye but also the surrounding tissues (the whole orbit), including muscle, bone, and vasculature. This intricate approach has the potential to deliver comprehensive and functional vision restoration when combined with nerve regeneration and other techniques. Rejection rates in VCAs are generally high due to the diverse tissue types involved, necessitating specialized immunosuppressive regimens to prevent rejection (12–14). Although immunological privilege has been discussed in the context of intraocular structures, it is unclear whether this would be observed during WET. The choroid, being highly permeable to cells and proteins (15), increases the likelihood that the immune privilege of the eye is compromised during transplantation. This exposure of delicate intraocular tissues, especially the retina, makes it more susceptible to immune-mediated damage (16), presenting a more complex immunological challenge than other forms of VCA. Such responses can culminate in the rejection and loss of the transplanted eye, underlining the intricacies involved in ensuring the success of WET (17).

Decades of research have greatly advanced our understanding of immunological responses in VCAs and solid organ transplants (18–22). However, the specific immune response to WET remains unknown. Crucial aspects such as the earliest indicators of immune rejection in WET, the types of rejection responses observed, the primary clinical signs and laboratory findings associated with rejection, and strategies for early diagnosis and monitoring are not yet fully understood. Equally important is differentiating between immune rejection and other causes of inflammation, such as infection, in WET recipients. This lack of detailed knowledge represents a significant gap in our ability to effectively diagnose, monitor, and manage immune responses in WET, underscoring the need for more targeted research in these areas.

Research on WET is in its early stages. Initial studies have successfully established animal and cadaver models for WET, showcasing its clinical feasibility, with a recent patient case further highlighting the potential for clinical application (23–26). Reviews on the technical feasibility and immunological considerations of WET provide further insight into the challenges and progress in this field (27, 28). The ultimate success of WET in a clinical setting hinges on a deeper understanding of its unique immunological responses. While the immunological characteristics specific to WET have not been extensively documented, insights can be gleaned from established mechanisms of immune responses to allogeneic organ transplantation. These mechanisms typically involve innate, cell-mediated, and antibody-mediated rejection. Additionally, ischemia-reperfusion injury (IRI), an inevitable aspect of vascularized organ transplantation, has been shown to exacerbate alloimmunity and rejection severity (29). During rejection, cytokines, chemokines, and complement components are actively involved in various stages of the immune response. These molecular changes can be detected through laboratory tests. Furthermore, VCA, including WET, offers the advantage of external observation of graft status and relatively accessible pathologic biopsy. The presence of corneal tissue in WET also suggests that corneal transplantation research could offer valuable insights for immune response studies in WET.

In our laboratory, we have developed a rat orthotopic vascularized composite whole eye transplantation model, which we refer to as ‘WET animals’ or simply ‘animals’ throughout this paper for simplicity. We have validated the blood supply and structural integrity of this model using non-invasive Optical Coherence Tomography (OCT) imaging. This study is dedicated to elucidating the complex alloimmune responses in WET by employing both syngeneic (Syn) and allogeneic (Allo) models for comprehensive comparative analysis. We have thoroughly characterized the timing, nature, and progression of immune responses in WET, concentrating on the sequence of innate, T cell-mediated, and antibody-mediated immune responses, as well as the related cellular and molecular mechanisms. Our work also includes an assessment of gross morphological changes in the grafts, detailed gene expression analysis, and serological evaluation of cytokines and chemokines. This approach offers a holistic view of the immune landscape in WET, spanning various levels of biological organization. The results of this study have led to the development of innovative diagnostic criteria and monitoring strategies for WET rejection. These groundbreaking findings have the potential to significantly advance the field of eye transplantation and hold promise for improving patient outcomes.

## 2 Materials and methods

### 2.1 Animals

Fourteen- to sixteen-week-old male Lewis (LEW) and Brown Norway (BN) rats (Charles River Laboratories, Wilmington, MA) were used. Animals were maintained and experiments completed under an Institutional Animal Care and Use Committee (IACUC) approved protocol in a specific pathogen–free environment at the University of Colorado.

### 2.2 Anesthesia and euthanasia

#### 2.2.1 Anesthesia

Both donor and recipient animals were anesthetized using an intraperitoneal injection of an anesthetic cocktail containing Ketamine (80 mg/kg), Xylazine (5 mg/kg), and Acepromazine (1.5 mg/kg), mixed with saline solution to achieve final concentrations of 40% Ketamine, 10% Xylazine, and 10% Acepromazine. Anesthesia was maintained by administering half-doses every 40 minutes initially, then every 30 minutes. If the animal began to awaken during the final stages of surgery, particularly after completing the anastomosis, 1-3% isoflurane was used as an alternative to additional injections. Throughout the procedure, animals were monitored every 5-10 minutes to ensure adequate anesthesia.

#### 2.2.2 Euthanasia

At the designated endpoint of the study, animals were euthanized using an intraperitoneal injection of a high-dose Ketamine/Xylazine/Acepromazine mixture (240 mg/kg Ketamine, 30 mg/kg Xylazine, and 3.5 mg/kg Acepromazine), followed by bilateral thoracotomy or exsanguination to ensure death. This method follows the dual-method euthanasia guidelines to ensure the animals are deeply anesthetized and unresponsive to stimuli prior to performing secondary procedures to confirm death.

### 2.3 Orthotopic whole eye transplantation

**Figure 1:**
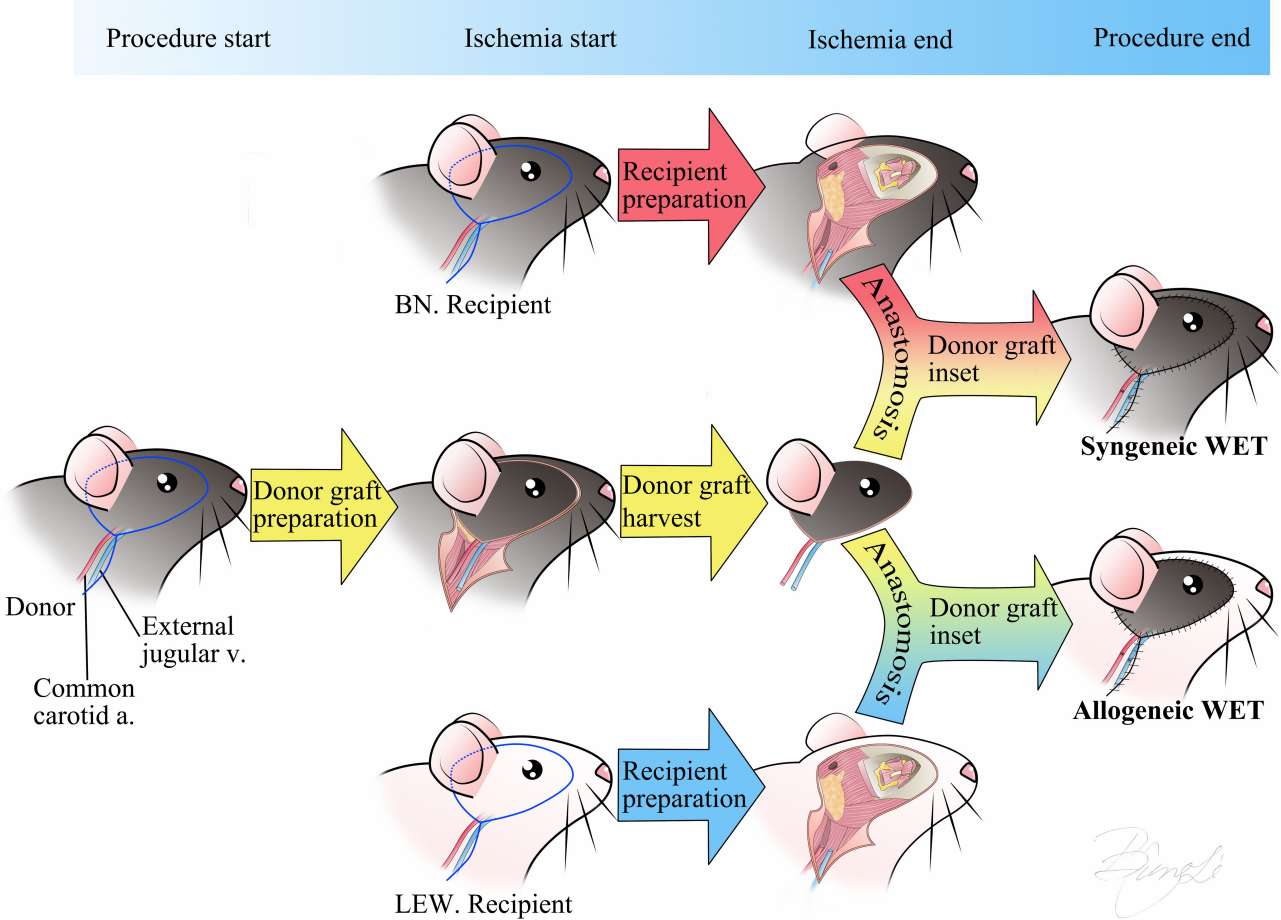
Schematic representation of the Syn/Allo whole eye transplantation procedure. The surgical incision design includes the skin around the eye and auricle with a linear extension for exposure of the common carotid artery and external jugular vein. The transplantation procedure involves three main steps: 1) donor graft preparation, 2) simultaneous donor graft harvest and recipient preparation, and 3) anastomosis of the donor and recipient arteries and veins, coaptation of the optic nerves, and suturing of the skin. Donor ischemia time starts at the point of donor graft harvest and ends at anastomosis finish. BN, Brown-Norway rat; LEW, Lewis rat; Syn, Syngeneic transplant; Allo, Allogeneic transplant; WET, Whole Eye Transplantation, v, vein; a, artery.

Orthotopic transplantation of the right eye and hemiface from donor BN rats to recipient BN or LEW rats was performed under a surgical microscope (Zeiss, Oberkochen, Germany). Following successful anesthesia, buprenorphine SR (1.2 mg/kg) was administered subcutaneously for sustained analgesia. The donor flap was prepared first, including the right eye, optic nerve, surrounding orbital contents, skin around the eye and auricle, and a portion of the cranial base to preserve intact blood circulation. The common carotid artery and external jugular vein were isolated and preserved as the vascular pedicle for the flap. Next, the recipient was prepared. The right eye of the recipient was enucleated, and the optic nerve, common carotid artery, and external jugular vein were isolated and prepared. The donor flap was then inserted orthotopically into the recipient site. End-to-end anastomoses of the donor and recipient common carotid arteries and external jugular veins were performed using 10-0 nylon sutures. The donor optic nerve end was precisely coapted to the recipient optic nerve end with 10-0 nylon sutures. Restoration of retinal blood supply was confirmed under the surgical microscope by observing perfusion of the fundus vessels. Finally, the muscles and skin were closed with interrupted 5-0 nylon sutures. Detailed schematics of the surgical procedures for syngeneic (Syn) and allogeneic (Allo) WET are provided in **Figure 1**. The procedure resulted in an average intraoperative ischemia duration of 82 ± 5 minutes and a total surgical time of 145 ± 10 minutes.

### 2.4 Study design and groups

Animals were allocated into the following groups:

(i) longitudinal assessment of rejection and survival group (Allo= 5 and Syn= 5); (ii) cross-sectional assessment of rejection group (Allo= 36, Syn= 21, Naïve= 9), given time-points: POD2, 4, 5, 6, 8. In cross-sectional groups, a total of 30 WET models from the Allo group and 15 WET models from the Syn group were used for histopathology study, with 6 animals per timepoint in the Allo group and 3 animals per timepoint in the Syn group. Twelve WET models were used for the qPCR study, with 3 animals in the Allo group and 3 animals in the Syn group on both POD2 and POD5, respectively (**Supplementary Table 1**).

### 2.5 WET viability assessments and monitoring

**Figure 2:**
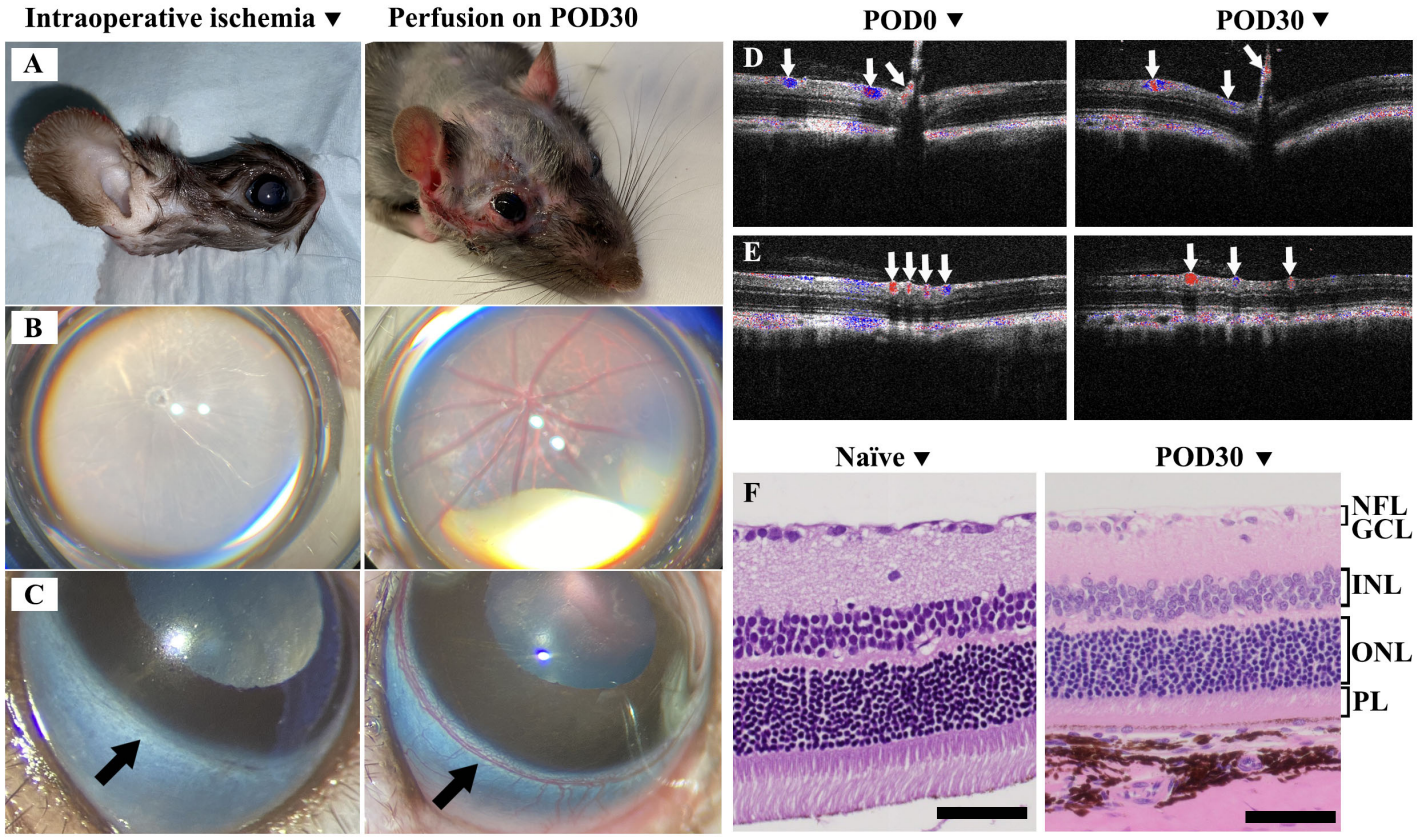
Evaluation of ischemic response and postoperative recovery in Syn WET. **(A–C)** showed the ischemia in the graft skin, retinal, and limbal vasculature during surgery (Left) and demonstrated good perfusion at 30 days after Syn WET (Right). **(D, E)** showed Doppler OCT images, illustrating blood flow in central retina **(D)** and peripheral retina **(E)** before and 30 days after Syn WET surgery; blue and red colors indicate venous and arterial flow (white arrows), respectively. **(F)** presents Hematoxylin and Eosin (H&E) stained sections comparing a naïve retina with a retina from a Syn animal 30 days post-WET. The comparison reveals no significant changes in the retinal structure, except for some thinning observed, especially in the outer nuclear layer (ONL) and photoreceptor layer (PL). Scale bar: 50 μm in **(F)**.

In rats, the anterior segment of the eye primarily receives its blood supply from the iris and ciliary arteries, which are connected to the limbal artery. The posterior segment benefits from a dual blood supply system. While the central retinal artery primarily perfuses the inner layers of the retina, the choroidal vasculature, which is perfused by the posterior ciliary arteries, provides additional vital support. The posterior ciliary arteries also supply the anterior portion of the optic nerve, underscoring their critical role in the overall vascular architecture of the eye (30). Therefore, we tailored our examination of ocular perfusion to these anatomical considerations. During surgery, we meticulously assessed ocular blood perfusion by examining the retinal and limbal vessels under the surgical microscope. Additionally, we evaluated the graft skin perfusion by observing the color changes during ischemia and reperfusion phases (**Figures 2A–C** Left).

Postoperative blood supply examination: After surgery, graft perfusion was monitored through direct observation of skin color, while retinal vessel perfusion was examined using OCT and Doppler OCT (Bioptigen, Durham, NC), along with a binocular indirect ophthalmoscope (HEINE Optotechnik, Gilching, Germany). Limbal vasculature was evaluated via the surgical microscope. Representative images in **Figures 2A–C** right panels illustrate skin, retinal, and limbal vessel perfusion on POD30, Panels D and E display Doppler OCT images, with panel D depicting the central retina and panel E showcasing the peripheral retina, both used to illustrate the arteries and veins before and 30 days after WET.

Postoperative intraocular pressure (IOP) and structural examination: IOP was measured using an iCare TONOLAB tonometer (Tonovet, Vantaa, Finland), with normal IOP considered to be between 7.28 and 26.98 mmHg (31). The binocular indirect ophthalmoscope and OCT were used to examine the cornea, iris, lens, and retinal layers of the transplanted eye. Ocular histopathology was performed on longitudinal samples at 30 days post-surgery to assess retinal layer integrity, compared with naïve eye samples (**Figure 2F**).

**Figure 3:**
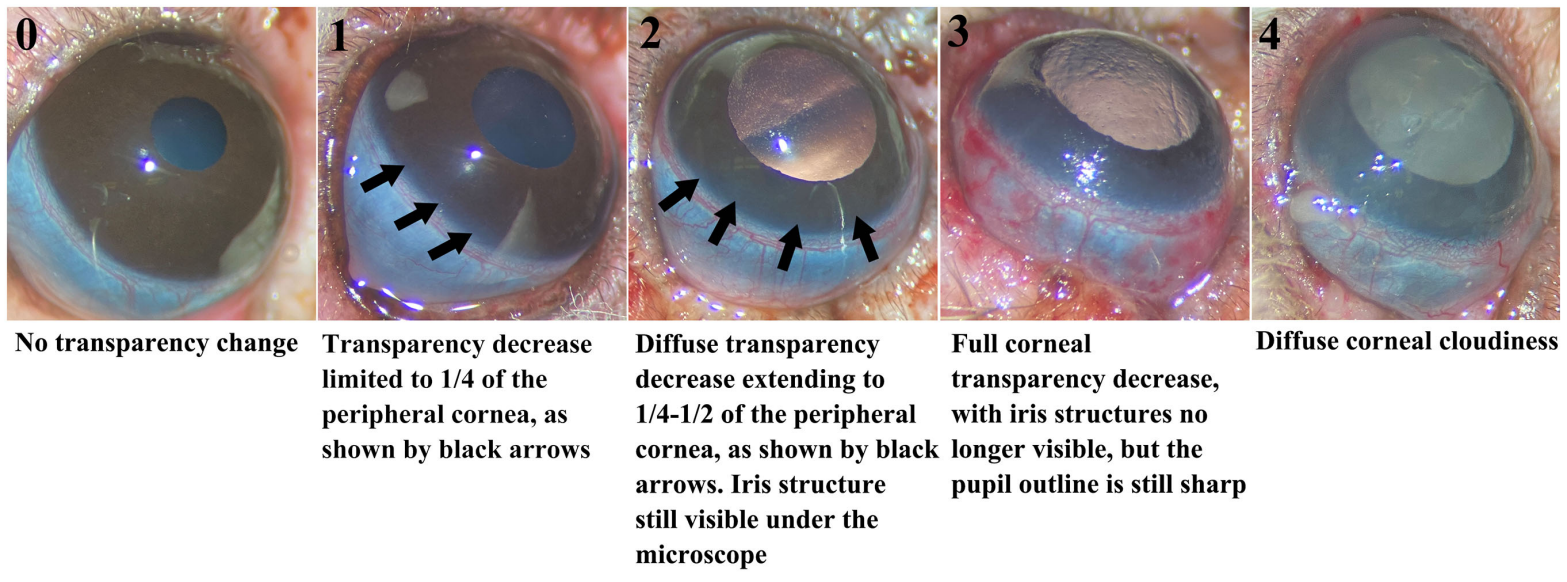
Modified corneal score system for grading rejection. The five images illustrating corneal transparency changes for grades 0-4. Changes for scores 1 and 2 are indicated by black arrows in the images.

Postoperative ocular rejection assessment: We diligently monitored WETs daily using a modified corneal scoring system to preliminarily assess eye rejection. The scores ranged from 0 to 4 based on corneal opacification: 0 = no transparency change, 1= slight transparency change limited to ¼ of the peripheral cornea, 2 = diffuse transparency decrease extending to ¼ to ½ of the peripheral cornea, but with iris structure still visible, 3 = full corneal transparency decrease, with iris structures no longer visible but the pupil outline remaining sharp, and 4 = Diffuse corneal cloudiness. Grafts receiving a score of 3 or higher were classified as rejected (see **Figure 3**).

Corneal thickness assessment: To quantify post-transplantation changes in corneal thickness, OCT was employed, with a specific focus on the central corneal thickness. Baseline measurements were established for each animal prior to transplantation (on post-operative day 0, POD0). These baseline values were then compared to measurements taken on subsequent post-operative days (POD*x*). The relative changes in corneal thickness were calculated using a fold change formula:


$$\;corneal\;thickness\;fold\;change_{PODx}=\frac{thickness_{PODx}}{baseline\;thickness_{POD0}}$$

**where** x=0, 2, 4, 5, 6, 8

Postoperative skin rejection assessment: Skin were monitored daily and scored for rejection based on physical examination using the following scale: grade 0 (no rejection), grade I (edema), grade II (erythema and edema), grade III (epidermolysis), and grade IV (ulceration, exudation, and necrosis). Grafts were considered rejected when displaying signs of progressive grade III rejection (32).

### 2.6 Gene expression and quantitative real-time polymerase chain reaction

The mRNA (messenger ribonucleic acid) expression profiles of rejection markers were evaluated in the eye on POD2 and POD5 for both Allo and Syn WET models. Additionally, gene expression was initially normalized to naïve control samples, setting a baseline to determine relative upregulation or downregulation within each group. Eye samples were collected and snap frozen after perfusion with phosphate-buffered saline solution. Total mRNA was extracted from samples using TRIzol™ Reagent (ThermoFisher Scientific, Waltham, MA), purified using the Qiagen RNeasy Plus Mini kit (Qiagen, Germantown, MD), and quantified using a NanoDrop 2000 spectrophotometer (ThermoFisher Scientific). For each reverse transcription reaction, 800 ng of high-quality RNA was converted to complementary deoxyribonucleic acid (DNA) using the RT^2^ First Strand Kit (Qiagen). Quantitative real-time polymerase chain reaction (qRT-PCR) was then performed using RT² SYBR Green ROX qPCR Mastermix (Qiagen) with the RT² Profiler™ PCR Array Rat Transplant Rejection plate (Qiagen) according to manufacturer’s instructions. Relative fold changes of the mRNA expression of 84 genes were calculated from duplicate Ct values. Normalization was performed using Hypoxanthine Phosphoribosyltransferase 1 (*HPRT1*) as the reference gene, selected based on its consistent expression across all experimental conditions. The selection of HPRT1 was further supported by the Automatic Selection from HKG Panel in the QIAGEN GeneGlobe Data Analysis Center, which evaluates reference gene stability using a set of housekeeping genes. The software automatically identified HPRT1 as one of the most stable reference genes, and its geometric mean was used as the normalization factor. Fold changes were calculated using the ΔΔCt Method, and results were averaged and reported. Naïve BN whole eyes served as untreated controls. The gene listing was as follows: *ITGAE*, *GZMA*, *IL10*, *CCR7*, *CXCl9*, *GZMB*, *STAT4*, *CD80*, *CXCl10*, *IL2RA*, *CD28*, *IL12B*, *MS4A1*, *CCR2*, *TLR9*, *MMP7*, *COL1A2*, *IL5*, *CXCL11*, *CCL2*, *CD8A*, *PRF1*, *NOS2*, *IL12A*, *IFNG*, *CCR3*, *CXCR4*, *MMP1B*, *PSMB9*, *CASP1*, *TNFSF10*, *STAT6*, *THBS2*, *TNF*, *CSF2*, *CD40*, *CCL5*, *CXCR3*, *BMP7*, *THBS1*, *CCR5*, *ADAM17*, *IL2*, *IL4*, *MMP1*, *NFKB1*, *CTLA4*, *CCL3*, *TLR4*, *TGFB2*, *FASLG*, *CCL4*, *TGFB3*, *CD40LG*, *IL16*, *CD86*, *ITGAM*, *CCL11*, *CD44*, *STAT1*, *CXCR2*, *IL13*, *CTGF*, *ITGA2*, *IL3*, *CX3CR1*, *CASP8*, *CD14*, *FAS*, IL1B, *MMP9*, *ICAM1*, *TAP1*, *TLR3*, *TGFB1*, *VEGFA*, *CASP3*, *PECAM1*, *C4A*, *VCAM1*, *C3*, *MMP2*, *IL6*, and *TIMP1*. The -ΔΔCt data was used to generate the heatmap for data visualization.

### 2.7 Histological staining and light microscopy analysis

Skin and transplanted eye samples were obtained from the cross-sectional group at their given time-points (POD2, 4, 5, 6, 8), or from longitudinal group at POD30. Samples were fixed in 10% neutral buffered formalin, paraffin-embedded, sectioned at 5μm, and stained with hematoxylin and eosin (H&E) for microscopic examination of tissue architecture and mononuclear cell infiltration.

To determine the locations of immune cells within the transplanted eye, ocular tissues were stained with anti-CD3 and anti-Myeloperoxidase (MPO) antibodies for immunohistochemistry (IHC). Five-micron thick paraffin sections were prepared for immunodetection and stained with CD3 antibody (Abcam, ab16669, Cambridge, UK) at a dilution of 1:500. Sections required modest antigen retrieval in 10 mM sodium citrate, pH 6.0, with 0.1% Tween 20, for 10 minutes at 110°C in the NxGen Decloaker (Biocare Medical, Concord, CA) with a 10-minute cool down. Immunodetection was performed at room temperature in a humidity chamber. Nonspecific proteins were blocked with 2.5% normal goat serum for 20 minutes. The primary antibody was incubated for 60 minutes and then detected with Rabbit ImmPress Alkaline Phosphatase detection system from Vector Labs (cat# MP-5401, Burlingame, CA) for 30 minutes. Immune complexes were visualized with ImmPACT Vector red substrate for 20 minutes (cat# SK-5105; Vector Labs). All sections were counterstained in Harris hematoxylin for 2 minutes, blued in 1% ammonium hydroxide, dehydrated in graded alcohols, cleared in xylene, and covered glass mounted using synthetic resin. To confirm the specificity of the immunostaining and account for potential non-specific binding, we included secondary antibody-only controls in the IHC protocol. This involved omitting the primary antibody and using only the primary antibody diluent. No detectable staining occurred, ensuring that the observed immunostaining was specific to the primary antibody. Five-micron thick paraffin sections were prepared for immunodetection and stained with MPO antibody (cat# ab208670, Abcam, Waltham, MA) at a dilution of 1:1000. Sections required antigen retrieval in Borg Decloaker pH 9.5, with 0.1% Tween 20, for 10 minutes at 110°C in the NxGen Decloaker (Biocare Medical, Concord, CA) with a 10-minute cool down. The subsequent steps were the same as CD3 staining. Negative controls to confirm the specificity of the immunostaining included omission of the primary antibody incubation step in the IHC protocol and substitution with the primary antibody diluent.

Ocular and skin histopathology samples were reviewed retrospectively in a blinded fashion by a board-certified pathologist specializing in ophthalmic pathology and a veterinary pathologist, respectively. Slides were observed under the Nikon Eclipse 55i microscope and representative micrographs were taken at different magnifications. Figures were assembled in Adobe Photoshop 2020.

### 2.8 Histological skin rejection assessment

The modified Banff VCA criteria were utilized for grading skin rejection (33). Grade 0 indicates no inflammatory activity. Grade 1 is characterized by mild dermal inflammation without any epidermal involvement. Grade 2 exhibits pronounced dermal inflammation, further differentiated into 2A (epidermis not involved) and 2B (epidermis affected). Grade 3 signifies severe inflammation, subdivided into 3A (isolated keratinocyte necrosis) and 3B (segmental full-thickness epidermal necrosis with some intact epidermal areas). Grade 4 is marked by diffuse full-thickness epidermal necrosis, indicating the most severe level of rejection.

### 2.9 Serum cytokine/chemokine analysis

In the cross-sectional assessment group, whole blood samples were collected at the time of euthanasia by cardiac puncture from animals euthanized at POD2, 4, 5, 6, and 8. For naïve controls, blood samples were drawn from LEW rats. Serum was extracted at 1400 rpm using Eppendorf Centrifuge 5801R (Hamburg, Germany) at room temperature for 10 min. Serum samples were used to test cytokine/chemokines by enzyme-linked immunosorbent assay (ELISA) and Luminex. A total of 22 cytokines and chemokines were assayed simultaneously by Luminex according to manufacturer instructions (Invitrogen™, Cytokine & Chemokine 22-Plex Rat ProcartaPlex™ Panel, Thermo Fisher Scientific, Waltham, MA). The cytokine/chemokines list was as follows. Cytokines: G-CSF (CSF-3), GM-CSF, IFN-γ, IL-1α, IL-1β, IL-2, IL-4, IL-5, IL-6, IL-10, IL-12p70, IL-13, IL-17A (CTLA-8), TNF-α. Chemokines: Eotaxin (CCL11), GROα (CXCL1), IP-10 (CXCL10), MCP-1 (CCL2), MCP-3 (CCL7), MIP-1α (CCL3), MIP-2, RANTES (CCL5). Analysis was done by Luminex Magpix^®^ system (Luminex, Austin, TX, USA). ELISA analysis for CXCL11 and CXCR3 expression was also performed on serum using CXCL11 and CXCR3 rat ELISA kits according to manufacturer’s instructions (Aviva Systems Biology, San Diego, CA).

### 2.10 Serum donor specific antibody test

Blood samples from longitudinal assessment group Allo recipient rats (LEW) were collected *via* tail vein before surgery and after surgery on POD2, 4, 5, 6, and 8. Serum was extracted at 1400 rpm using Eppendorf Centrifuge 5801R (Hamburg, Germany) at room temperature for 10 min. Dithiothreitol (DTT) was optionally used to reduce the disulfide bonds in the IgM molecule and thus functionally inactivate IgM, making it possible to differentiate between the effects of IgG and IgM in a serum sample. In brief, an aliquot of 1M DTT (Invitrogen™, Carlsbad, CA) was thawed at room temperature, a 10mM solution of DTT was prepared by diluting 1M DTT 1/100 in Hanks Balanced Salt Solution with calcium and magnesium (HBSS, Invitrogen™, Carlsbad, CA). After mixing well, 25µL of 10mM DTT was added to 25µL of serum. This was incubated for 30 minutes in a 37°C water bath. The treated sample was used immediately in the assay. Naïve BN lymph nodes were harvested then processed to form single-cell suspensions. One million lymphocytes per milliliter were used to test serum DSA on different PODs. 10µL serum from different PODs, with or without DTT treatment, were incubated with lymphocyte for 30 minutes at 4°C and then stained with either anti-Rat IgM (A-21212) or IgG (A-11006) (Thermo Fisher Scientific, Waltham, MA) at 1:200 dilution plus 7-Aminoactinomycin D (7AAD) viability dye (Invitrogen™, Carlsbad, CA), respectively. Stained lymphocytes were analyzed on a Beckman cytometer (Beckman Coulter, Brea, CA) and results were analyzed using FlowJo (Ashland, OR).

### 2.11 Statistical analysis

Statistical analyses were conducted using GraphPad Prism v8. Data were presented as means ± SD or means ± SEM, as appropriate for the distribution. To discern significant differences between experimental groups, the Wilcoxon rank sum test was applied to non-continuous variables, while independent t-tests or two-way ANOVA, followed by Tukey’s *post hoc* tests for multiple comparisons, were utilized for continuous variables, assessing both group and timepoint interactions. Repeated measures ANOVA was employed for repeated measurements to evaluate overall time and group effects, accompanied by Dunnett’s Multiple Comparisons. For the DEGs data, p-values were calculated based on a Student’s t-test of the replicate 2^ (-Delta CT) values for each gene in the naïve control and treatment (Syn and Allo) groups. Differences were considered significant at *P* < 0.05.

## 3 Results

### 3.1 Evaluation of the rodent model for WET

Within our longitudinal study group, three Syn WET animals were specifically evaluated to assess the restoration and long-term stability of blood supply, IOP, and structural integrity.

#### 3.1.1 Restoration of blood supply after WET

Intraoperative observations revealed complete ischemia in both the graft skin and the eye immediately after arterial and venous transection, as illustrated in **Figures 2A–C** (left panels). These images show ischemia in the skin, retina, and limbal vessels, respectively. Following vascular anastomosis, prompt reperfusion was noted. By POD 30, consistent and adequate perfusion in the transplanted eye and skin was confirmed, as depicted in **Figures 2A–C** (right panels), which display perfusion in the skin, retina, and limbal vessels, respectively. Additionally, Doppler OCT was employed to assess retinal blood flow both preoperatively (POD0) and at POD30, verifying the restoration of both central and peripheral retinal arterial and venous flow after WET, as evidenced in **Figures 2D, E**.

#### 3.1.2 Evaluation of intraocular pressure and ocular structural integrity

Throughout the postoperative period, IOP readings remained within the normal range (data not shown), demonstrating maintenance of ciliary body function. Postoperative assessments confirmed the maintenance of corneal transparency with intact epithelium, stroma, and endothelium. Lens clarity, iris epithelium integrity, functional contractility and dilation were also preserved.

OCT imaging (**Figures 2D, E**) and histological analyses of the SYN eye compared to a naïve eye (**Figure 2F**) revealed that the overall structural integrity of the retinal layers was largely maintained 30 days post-surgery. While some thinning was observed in the outer nuclear layer (ONL), consistent with a reduction in the photoreceptor layer (PL), the inner nuclear layer (INL), retinal ganglion cell layer (GCL), and nerve fiber layer (NFL) also showed some cellular changes. However, the retinal structure as a whole remained intact. This highlights the potential of this model for further research in eye transplantation immunology studies without significant compromise to the overall retinal architecture.

### 3.2 Assessments of rejection-induced gross changes after WET

In this study, a total of 72 WET animals were established, with 44 in the Allo group and 28 in the Syn group. Viability assessments were conducted on POD2. Animals exhibiting signs of graft ischemia (such as pale skin and retina), significant corneal damage, loss of lens transparency, other ocular structural abnormalities, or poor postoperative conditions (including respiratory distress or dehydration) were excluded from further analysis. As a result, five animals (3 Allo and 2 Syn) were excluded due to labored breathing (3 Allo), dehydration and retinal ischemia (1 Syn), and failure to recover from anesthesia (1 Syn), leading to a surgical success rate of 93.1% (67 out of 72). Animals that passed the initial viability checks were then subjected to tailored postoperative monitoring and testing protocols based on their respective groups. The grouping details and the distribution and number of animals in each group are summarized in **Supplementary Table 1**.

#### 3.2.1 Decreased corneal transparency and increased thickness following Allo WET

**Figure 4:**
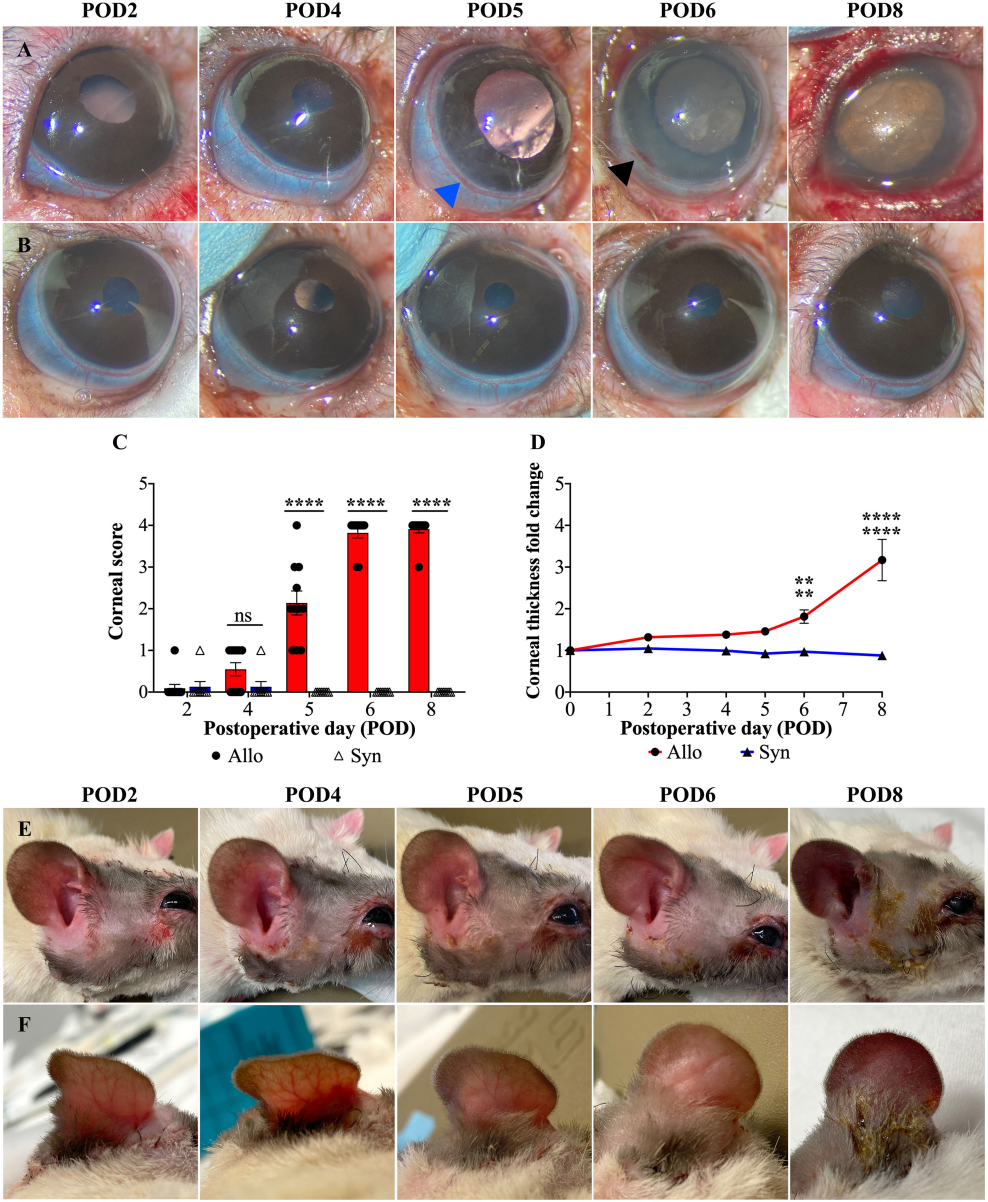
Postoperative gross changes in eye and skin following WET. **(A, B)** Representative images of corneal transparency and limbal vasculature in Allo and Syn animals over POD2 to 8 (POD2-8). In the Allo group, limbal vessels show hyperemia (blue arrowhead), patchy perilimbal subconjunctival hemorrhage (black arrowhead), and complete disappearance of visible vessel structure indicating severe damage. **(C)** Corneal rejection scores for Allo and Syn animals, with a significant increase in the Allo group from POD5 onwards (n=11 per time-point for Allo, n=8 per time-point for Syn), ‘ns’ denotes not significant. **(D)** The fold increase in corneal thickness reveals a significant thickening in Allo animals at POD6 and POD8 when compared to both baseline (POD0) and to the Syn animals at the same time points (n=5). **(E, F)** Macroscopic skin changes on the Allo animals, showing progression from rash to purple discoloration, with persistent edema noted throughout the observation period. For the comparison of corneal scores, differences between groups at each time point were compared using the Wilcoxon rank sum test. For corneal thickness, independent t-tests were utilized for between-group comparisons, and repeated measures ANOVA was employed to assess overall time and group effects, accompanied by Dunnett’s Multiple Comparisons. Significance levels are denoted as: ns, not significant; **P<0.01, and ****P<0.0001.

The Allo group exhibited a rapid and progressive decrease in corneal transparency between POD4 and POD6, in contrast to the Syn group, as shown in **Figures 4A, B**. From POD5 onwards, the Allo group showed significant differences in corneal transparency compared to the Syn group, consistently exhibiting signs of corneal rejection within 6 days post-transplantation (**Figure 4C**). Additionally, Allo animals displayed a marked increase in corneal thickness from POD6, with a 1.8-fold increase compared to the baseline (POD0), and a 2-fold increase compared to the Syn animals (**Figure 4D**). In contrast, Syn animals did not show significant changes in corneal thickness at any of the postoperative time points assessed.

#### 3.2.2 Limbal vasculature damage in Allo WET

The rat limbus is characterized by a circumferential vascular ring, comprising a solitary artery and a venous plexus, which is readily identifiable due to its superficial location (34) (as indicated by the blue and black arrowheads in **Figure 4A**). Hyperemia in the limbal vasculature was observed in both groups from POD2 to POD5. However, most Syn animals exhibited recovery by POD6 (5/6), while Allo animals exhibited a progression to patchy perilimbal subconjunctival hemorrhage after POD4 or POD5 (**Figure 4A**, blue arrowhead), and diffuse hemorrhage was observed on POD6 or POD8 (**Figure 4A**, black arrowhead).

#### 3.2.3 Lack of distinct stages in graft skin changes following Allo WET

Unlike typical stages observed in VCAs, Allo WET graft skin appearance changes were not distinctly stageable. Initially, all Allo grafts showed consistent edema from POD2 to POD5, without significant differentiation. However, by POD6, signs of advanced rejection, such as skin thickening and darkening, were observed in the majority of Allo animals (7/11). By POD8, all Allo animals exhibited conclusive rejection stages marked by skin ulceration and exudation. In contrast, the Syn group showed initial edema that resolved by POD6, indicating a different postoperative trajectory (**Figures 4E, F**).

### 3.3 Upregulation of rejection-related genes in Allo versus Syn transplanted eyes

**Figure 5:**
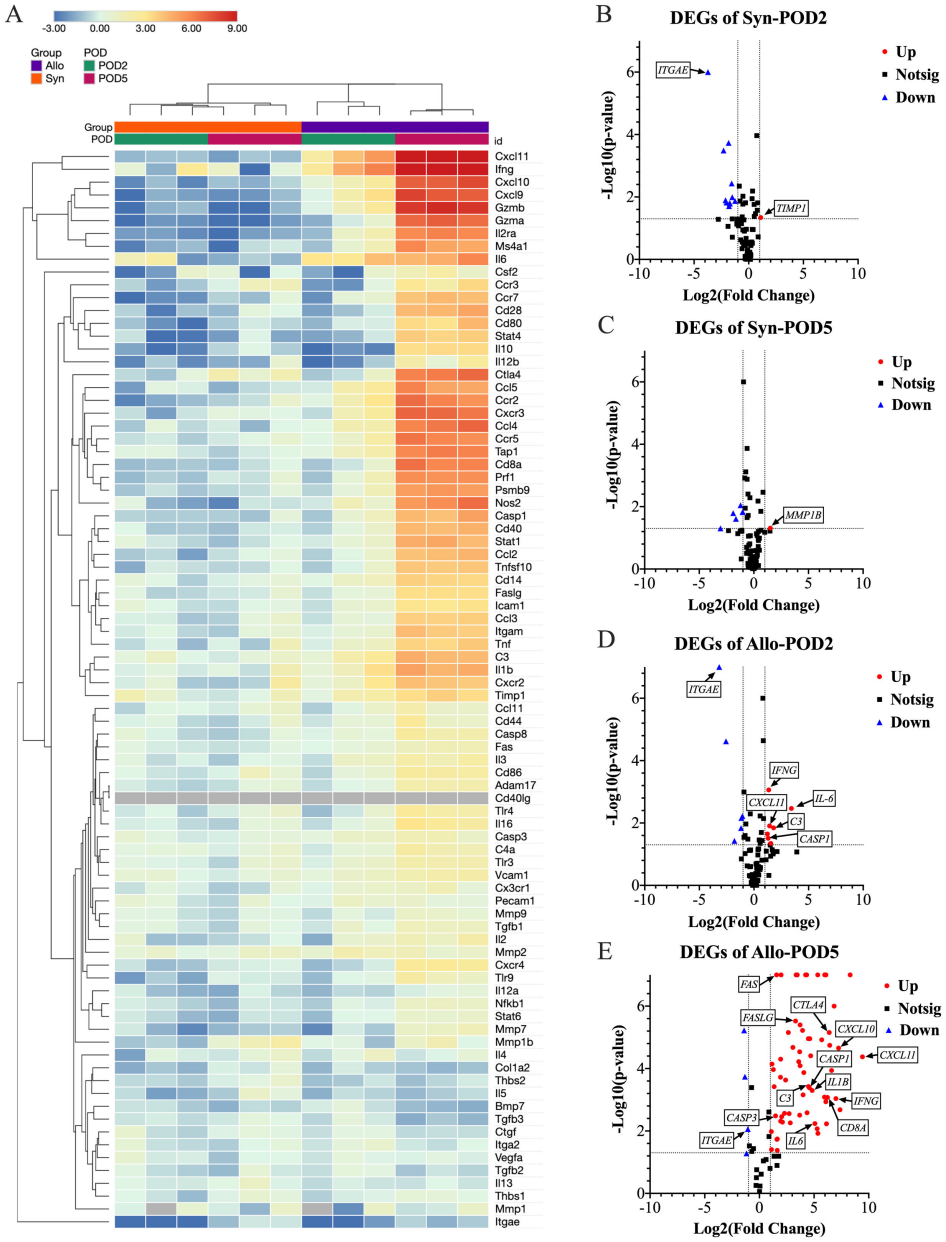
Differential gene expression analysis in eye samples post-WET. **(A)** This clustergram presents a heatmap with dendrograms depicting patterns of gene expression across different groups, with gray indicating genes not detected. The -ΔΔCt data was used to generate the heatmap for data visualization. At POD5, the Allo group exhibits a marked increase in gene expression. **(B–E)** Volcano plots delineate the significance and magnitude of gene expression changes, with key genes spotlighted. The volcano plot combines a p-value statistical test with the fold regulation change enabling identification of genes with both large and small expression changes that are statistically significant. The p values are calculated based on a Student’s t-test of the replicate 2^ (- Delta CT) values for each gene in the naïve control group and treatment (Syn and Allo) groups. (n=3, Fold change >2, P<0.05; Up, increased gene expression; Down, decreased gene expression; Notsig, not significant). The Syn group displays slight transcriptional increases with *TIMP1* at POD2 and *MMP1B* at POD5. In the Allo group at POD2, modest upregulation is noted in IL6, *C3, IL5, CXCL11, IFNG, CASP1*, and *GZMB*. By POD5, this upregulation intensifies, with 62 out of 84 rejection-related genes significantly increased. Notably, six of the initially upregulated genes show dramatic rises: *CXCL11* surges from a 2.68-fold to a 688.63-fold increase, *GZM*B from 2.35 to 315.69-fold, *IFNG* from 2.55-fold to 127.76-fold, *IL6* from 10.77-fold to 33.81-fold, *C3* from 3.48-fold to 22.54-fold, and *CASP1* from 2.46-fold to 23.67-fold. *IL1B*, a *CASP1* substrate, also significantly increases to 28.34-fold, without initial upregulation at POD2. Additionally, *CXCL9, CXCL10*, and *GZMA*, which were not upregulated at POD2, exhibit substantial increases exceeding 100-fold by POD5. Concurrently, *ITGAE* (*CD103*) is significantly downregulated approximately 10-fold, in both Allo (0.11-fold change) and Syn (0.08-fold change) animals at POD2, with a slight decrease persisting in the Allo group at POD5 (0.48-fold change). These significant gene expression shifts underscore the immune response dynamics following transplantation.

Elucidating rejection in transplantation involves understanding the dynamics and mechanisms of gene expression changes associated with the rejection process. The heatmap depicted in **Figure 5A**, illustrates the gene expression changes for both groups with respect to the naïve baseline, highlighting significant differences in the gene expression profiles associated with rejection between the two groups. Additionally, the volcano plots in **Figures 5B–E**, further delineated the differentially expressed genes in the Allo and Syn groups at POD2 and POD5, with a focus on significant changes. In the Syn animals, the expression levels of the 84 monitored rejection-related genes largely remained stable. At POD2, only the *TIMP1* gene showed upregulation, at a modest 2.14-fold increase. By POD5, the *MMP1B* gene was the sole gene exhibiting upregulation, at 2.84-fold. Conversely, Allo animals at POD2 displayed upregulation in seven genes, including *IL6, C3, IL5, CXCL11, IFNG, CASP1*, and *GZMB*, with *IL6* showing the most significant increase at 10.77-fold. The complement component *C3* followed at 3.48-fold, with the remaining genes experiencing increases between 2 to 3-fold. By POD5, a surge in gene expression was observed, with 62 out of the 84 genes upregulated. Specifically, six genes, including *CXCL9, CXCL10, CXCL11, IFNG, GZMA*, and *GZMB*, were highly upregulated, each by more than 100-fold, with *CXCL11* exhibiting the most pronounced increase at over 600-fold. Additionally, ten genes, including *CXCR3, CCL4, CTLA4, CD8A, CCR2, TAP1, CCR5, IL2RA, NOS2*, and *PRF1*, demonstrated moderate upregulation, with a 50 to 100-fold rise. Within the upregulated genes, those related to apoptosis such as *CASP3, CASP8, FAS*, and *FASLG* were all upregulated but to a lesser extent, with fold changes of 2.78, 4.30, 2.97, and 9.90 respectively. Of particular interest, *CASP1* (*Caspase-1*), a key enzyme in pyroptosis, was upregulated at 2.46-fold at POD2 and 23.67-fold at POD5. Its substrate, the pro-inflammatory cytokine *IL1B*, also saw a substantial upregulation to 28.34-fold at POD5, although it did not show an increase at POD2. Another notable observation was the significant downregulation of *ITGAE* (*CD103*) in both Allo (0.11-fold change) and Syn (0.08-fold change) animals at POD2, with a slight decrease persisting in the Allo group at POD5 (0.48-fold change).

### 3.4 Temporal and spatial patterns of immune cell infiltration in WET

#### 3.4.1 Absence of specific histological abnormalities in Syn WET eye

In Syn WET eyes, histological examination revealed all ocular tissues to be viable, without any detectable signs of cell death, mirroring the observations in naïve controls (data not shown). H&E staining, complemented by MPO and CD3 immunohistochemistry, did not reveal any significant deviations from the naïve baseline. Specifically, MPO staining showcased a distribution of scattered mast cells within the limbal conjunctiva, choroid, and orbital soft tissues in Syn WET animals that was akin to that in naïve eyes, thereby indicating the maintenance of normal cellular distribution post-transplantation. Similarly, the examination of retinal layers showed preserved cellularity comparable to naïve eyes. The absence of CD3 staining further confirmed a lack of T cell infiltration, aligning with the naïve condition. Nuclei were occasionally observed within the peripheral segments of photoreceptors that were negative for both CD3 and MPO markers in both groups potentially reflecting migration of photoreceptor precursors or cells undergoing mitotic cycling. This is supported by the documented oscillatory movement of photoreceptor nuclei between apical and basal positions during cell division (35, 36).

#### 3.4.2 Early inflammatory onset in extraocular tissues in Allo WET eye

**Figure 6:**
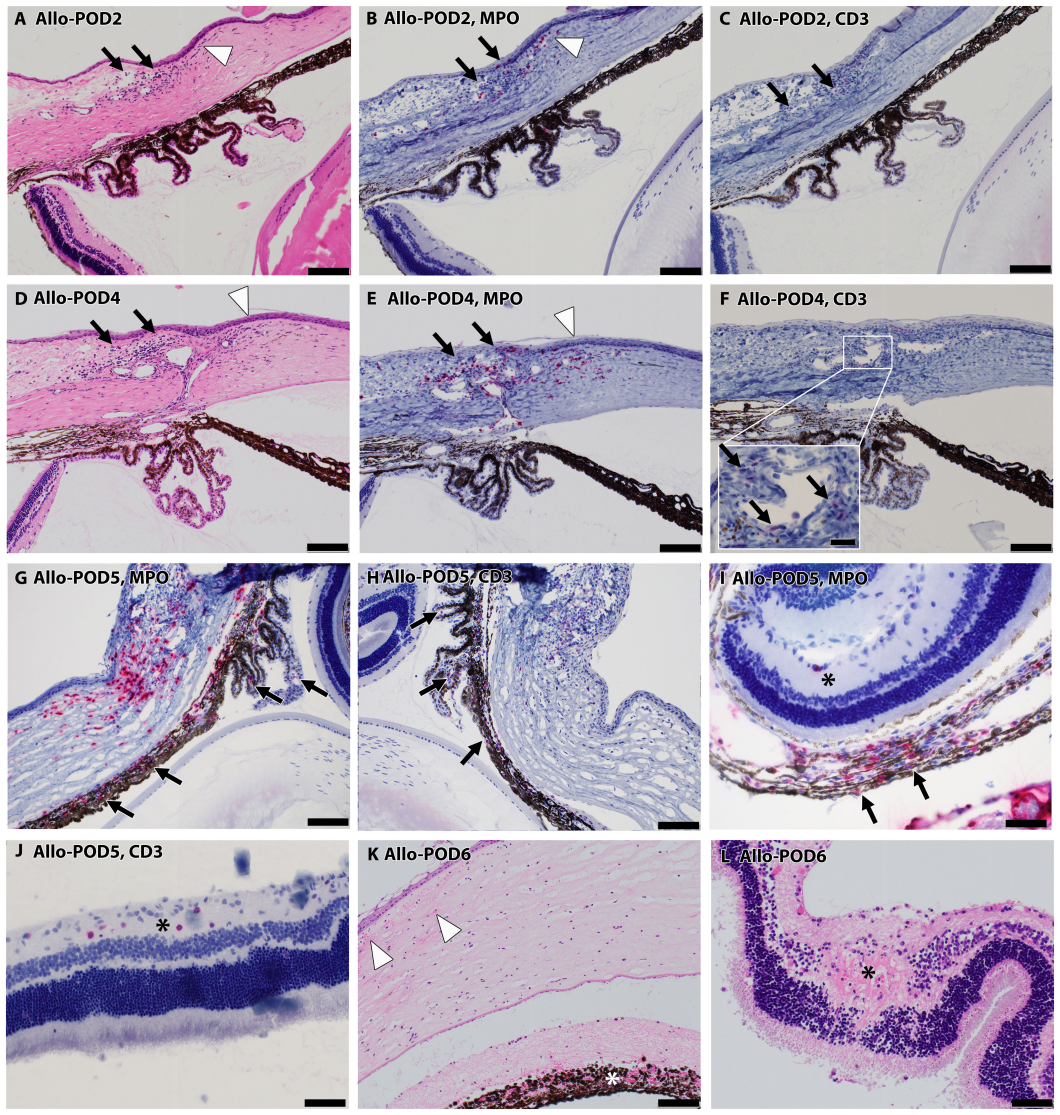
Progressive inflammatory infiltration in Allo WET over time. **(A–F)** depict escalating mononuclear inflammatory cell presence in the conjunctiva (black arrows), and peripheral cornea (white arrowhead) on POD 2 **(A–C)** and 4 **(D–F)**. The majority of these cells were MPO+ **(B, E)**, interspersed with CD3+ lymphocytes (**C, F** inset). **(G, H)** show increased MPO+ and CD3+ staining in iris and ciliary body indicated by black arrows on day 5, alongside advancing infiltration in the conjunctiva, limbus, and peripheral cornea. **(I, J)** show increased cellularity in the nerve fiber layer (asterisks) at POD5, comprising predominantly CD3+ **(J)** over MPO+ mononuclear cells **(I)**, despite substantial MPO+ infiltration in the choroid (**I**, black arrows). By POD 6, tissue necrosis becomes evident in some cases **(K)**, characterized by corneal edema, acute inflammation (white arrowheads), and necrosis, along with necrosis in the iris (asterisk) and retina (**L**, asterisk). Scale bars: **(A–H)**, K-100 μm; Inset F-20 μm; **(I, J)**, L-50 μm.

Extraocular tissues, comprising structures surrounding the eye such as the conjunctiva, lacrimal gland, and adipose tissue, exhibited inflammation onset as early as POD2, with an uptick in both MPO+ and CD3+ cells persisting through POD8. The adipose tissue and lacrimal gland exhibited more significant inflammation compared to the extraocular muscles. By POD2, four out of six samples showed predominantly mononuclear inflammation in the conjunctiva, all testing positive for MPO+ cells, and three samples presented sporadic CD3+ cells, as depicted in **Figures 6A–C** (black arrows). By POD4, this inflammation had intensified, showing a consistent moderate level characterized by a higher prevalence of MPO+ over CD3+ cells in all samples, shown in **Figures 6D–F** (black arrows).

#### 3.4.3 Progressive inflammation in peripheral cornea and choroid in Allo WET eye

Peripheral corneal inflammation was evident in half of the samples by POD2 H&E staining (**Figure 6A**, white arrowhead), with MPO+ cell elevation noted in two samples (**Figure 6B**, white arrowhead) and CD3+ cell presence in one. This inflammation was more marked by POD4, where MPO+ cells were more dominant, as seen in **Figures 6D, E** (white arrowheads). In the choroid, mild MPO+ inflammation was observed in three samples at POD2, escalating to involve all samples by POD4, predominantly featuring MPO+ over CD3+ cells.

#### 3.4.4 Delayed inflammatory response in intraocular tissues and central cornea in Allo WET eye

**Figure 7:**
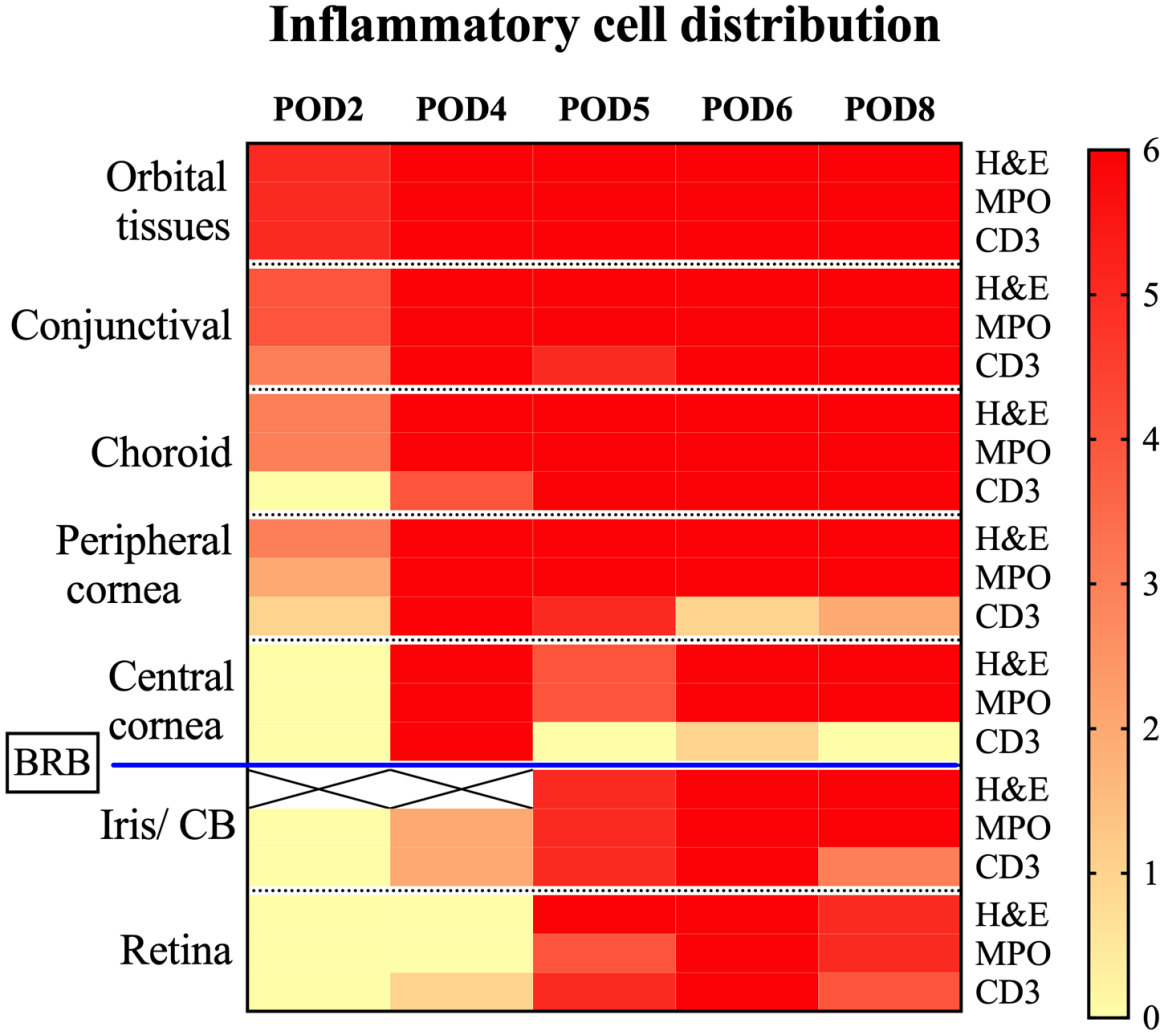
Heatmap depicting inflammatory cell distribution in Allo transplanted eyes post-WET. This heatmap displays the presence of inflammatory cells identified by H&E, MPO, and CD3 staining across different tissues after Allo WET. It covers a range of post-operative days (POD2 to POD8), with each column corresponding to a specific day. The rows categorize different tissue types and stainings. The color intensity on the heatmap indicates the number of samples (out of 6) exhibiting positive inflammation for each marker, ranging from 0 (no inflammation sample) to 6 (inflammation in all samples). White squares marked with an ‘X’ denote areas where dense pigmentation hindered reliable inflammation assessment via H&E staining. The blood-retina barrier (BRB) is noted, demarcating the regions outside from those within the barrier. CB, ciliary body; BRB, blood-retina barrier.

Intraocular tissues, encompassing structures behind the blood-retinal barrier (BRB) such as the iris, ciliary body, and retina, demonstrated a delayed inflammatory response. In our study, the iris and ciliary body showed initial signs of cell infiltration by POD4, as evidenced in one case with confirmed MPO+ and CD3+ staining. This infiltration became more widespread by POD5, as depicted in **Figures 6G, H** (black arrows). By POD6, cell infiltration was observed in all samples. At this stage, severe uveal necrosis accompanied by neutrophils was evident, as marked by the white asterisk in **Figure 6K**. Retinal changes began with the detection of CD3+ cells in a single sample by POD4, which escalated to widespread cell infiltration. By POD5, four samples displayed intermittent MPO+ mononuclear cells, and five samples exhibited mild CD3+ lymphocyte infiltration, marked by a black asterisk in **Figures 6I, J**. By POD6, infiltration by both cell types became pervasive, culminating in retinal necrosis and hemorrhage, as detailed in **Figure 6L** (black asterisk). The central cornea began showing inflammatory cells from POD4 in all samples, with a predominance of MPO+ cells and less frequent CD3+ cell detection. By POD6, necrosis, indicated by white arrowheads in **Figure 6K**, had become apparent. Additionally, **Figure 6I** offers insight into choroidal inflammation, showing significant MPO+ staining (black arrows) and providing an integrative perspective on the progressive immune response in ocular tissues. A heatmap depicting the distribution of cell infiltration across all ocular tissues in the Allo group is presented in **Figure 7**.

#### 3.4.5 Evaluation of skin histopathology and rejection grading of WET graft skin

**Figure 8:**
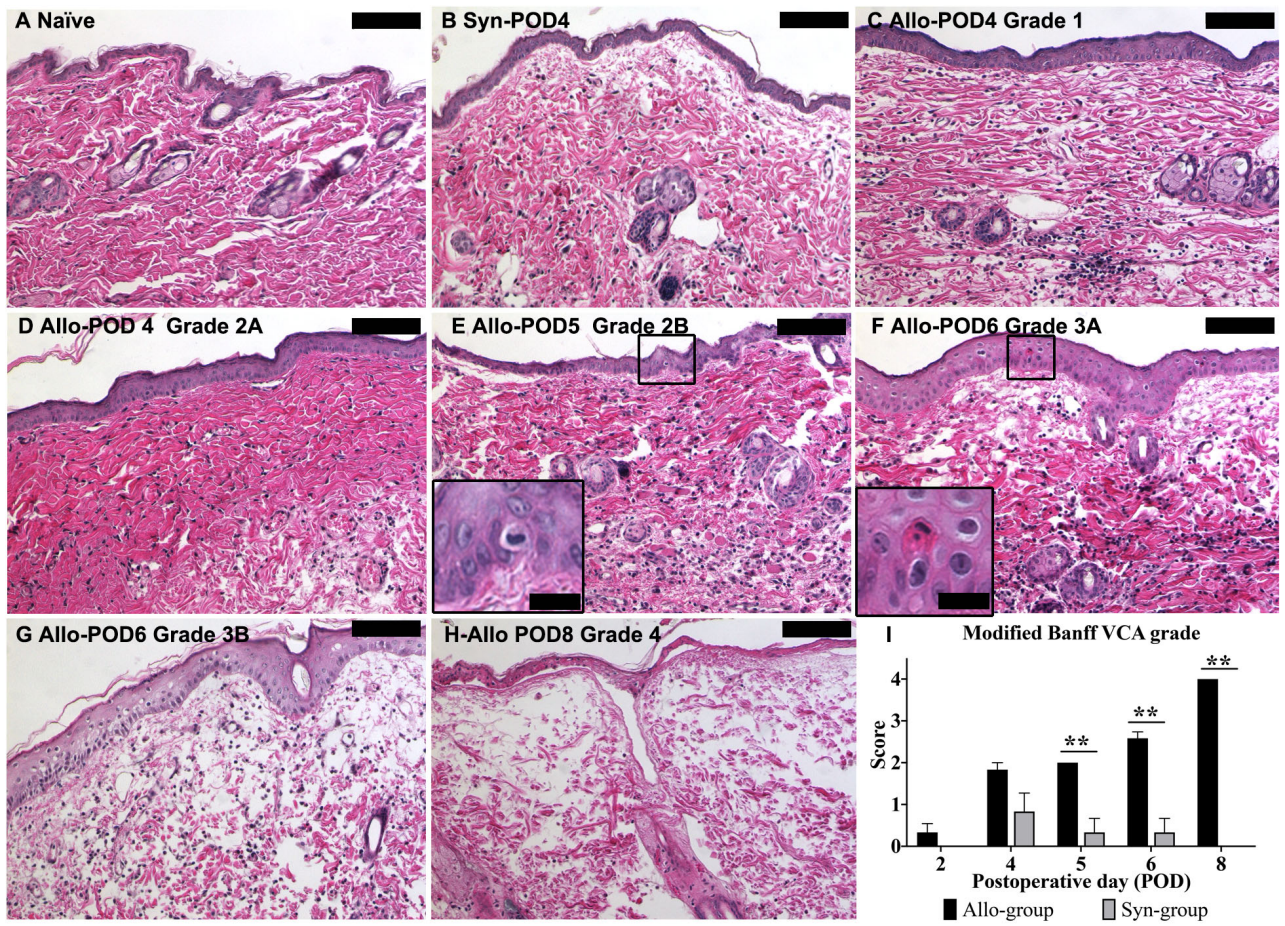
Histopathological evaluation of skin graft rejection using the modified Banff VCA criteria. **(A)** Control skin from a naïve animal showing normal histology. **(B)** Syn skin at POD4, with minimal cell infiltration indicative of Grade 1 rejection. **(C)** Allo skin at POD4, demonstrating Grade 1 rejection with mild inflammatory infiltration. **(D)** Allo skin at POD4, presenting with Grade 2A rejection featuring moderate infiltration without epidermal involvement. **(E)** Allo skin at POD5, with Grade 2B rejection characterized by inflammation reaching the epidermis (inset: higher magnification, 80X). **(F)** Allo skin at POD6, displaying Grade 3A rejection with isolated keratinocyte necrosis (inset: higher magnification, 80X). **(G)** Allo skin at POD6, showing Grade 3B rejection with multifocal epidermal necrosis (arrows). **(H)** Allo skin at POD8, exhibiting Grade 4 rejection with diffuse full-thickness necrosis of the epidermis. **(I)** Quantitative analysis indicates a statistically significant difference in rejection grades between Allo and Syn groups from POD5 onwards (n=6/time-point for Allo, n=3/time-point for Syn). Groups were compared by Wilcoxon rank sum test, and the significance level is denoted as **P < 0.01. All skin biopsies were retrospectively assessed in a blinded manner by a board-certified veterinary pathologist. Scale bars represent 100μm for the main images and 20μm for insets.

In contrast to the naïve skin sample (**Figure 8A**), Syn skin samples displayed inflammatory cell infiltration resembling grade 1 or 2A rejection seen in Allo samples. This inflammation in Syn samples began to decrease by POD5 and resolved significantly by POD8. We used a representative picture from POD4 (**Figure 8B**) to exemplify grade 1 rejection-type cellular infiltration in Syn, comparable to an Allo sample of the same grade (**Figure 8C**). In Allo samples, an escalation in inflammatory cell presence was noted from POD2, progressing to advanced-stage rejection changes. **Figures 8C–H** illustrate this progression in Allo animals, with increasing severity over time. The Modified Banff system scores were adapted to a modified numerical scale (0, 1, 1.5, 2, 2.5, 3, and 4) for ease of comparison. A significant divergence in skin rejection between the Allo and Syn groups was observed from POD5 onward, as illustrated in **Figure 8I**.

### 3.5 Serological Assessment after WET

#### 3.5.1 Correlation between serum CXCL10 and IFN-γ elevations and allograft rejection post-WET

**Figure 9:**
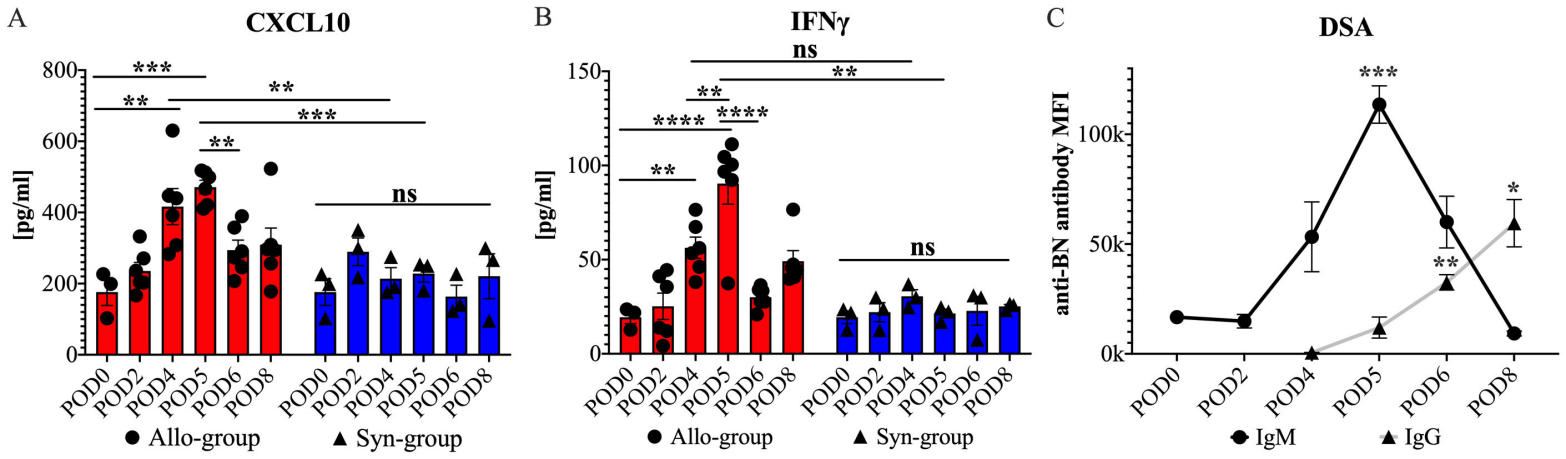
Serum cytokine, chemokine, and DSAs levels in cross-sectional and longitudinal studies post-WET. **(A)** shows a significant rise in serum CXCL10 levels on POD4 and 5 in the Allo group compared to the Syn group and baseline, with no difference between the two days, followed by a decrease on POD6. **(B)** illustrates an increase in serum IFN-γ levels on POD4 from baseline, without a significant difference from the Syn group, and a further increase on POD5 with significant differences noted both intragroup and between the Allo and Syn groups, and a subsequent decrease on POD6, with significance levels indicated (n=6/time-point for Allo, n=3/time-point for Syn). **(C)** demonstrates that donor-specific IgM levels increased by POD4, peaking at POD5 with significant differences compared to POD0. Meanwhile, donor-specific IgG levels became detectable by POD4 and showed significant increases by POD6 compared with the levels at POD4 (n=5). ‘ns’ denotes not significant; MFI stands for mean fluorescence intensity; ‘anti-BN’ refers to anti-Brown Norway. Cytokine and chemokine results were analyzed using two-way ANOVA to assess group and timepoint interactions, followed by Tukey’s *post hoc* tests for multiple comparisons. DSA levels were evaluated with repeated measures ANOVA, followed by Dunnett’s Multiple Comparisons. Significance levels are denoted as ns, not significant; *P<0.05, **P<0.01, ***P<0.001, and ****P<0.0001.

Our findings highlighted a significant increase in CXCL10 (IP-10) and IFN-γ serum levels in the Allo group after WET, compared with both the Syn group and baseline (naïve) levels. Specifically, in the Allo group, CXCL10 levels exhibited a significant rise by POD 4, relative to corresponding levels in the Syn group and baseline. Importantly, this elevated level was maintained on POD 5, with no significant difference observed between POD 4 and POD 5. In contrast, the Syn group did not exhibit any significant fluctuations in CXCL10 levels at any time point, either within the group or compared to baseline (**Figure 9A**). Similarly, IFN-γ levels in the Allo group showed a significant surge on POD5 compared to both the Syn group at the same time point and baseline levels. Interestingly, we observed a significant rise in IFN-γ levels on POD4 compared to baseline but not when compared to the Syn group (**Figure 9B**). The Syn group displayed no significant changes in IFN-γ levels at any point. For the remaining 22 cytokines and chemokines tested, no significant differences were observed between the Syn and Allo groups, although there were notable trends. These include a significant rise in IL-17A levels on POD4 in both groups, and a notable increase of certain cytokines and chemokines within the Allo group on POD4, while the Syn group showed minimal changes. Detailed serum results for these markers are presented in the **Supplementary Data** (**Supplementary Figure 1**).

#### 3.5.2 Antibody-mediated immune response in allograft rejection post-WET

Antibody-mediated rejection is recognized as a key factor in acute transplant rejection (37). To investigate its role following WET, we utilized flow cytometry to measure IgM and IgG levels in serum samples collected from day 0 to day 8 (POD0-8) in our longitudinal study. Our results showed a discernible increase in donor specific IgM, commencing on POD4 and significantly peaking on POD5. This was followed by the detection of donor specific IgG on POD6, the levels of which continued to rise until euthanasia on POD8 (**Figure 9C**). These findings suggested a contribution of antibody-mediated immune response during the acute rejection phase following WET.

## 4 Discussion

Advancing WET towards clinical application necessitates a thorough analysis of host responses following transplantation. Our study primarily focused on characterizing the gross morphological, genetic, histological, and serological alterations following WET. These observations are pivotal in constructing strategies to mitigate alloimmune reactions and in establishing diagnostic benchmarks crucial for monitoring and managing post-transplant outcomes. Laying a foundational framework for future investigations into the underlying immunological mechanisms.

### 4.1 Feasibility and rationality of our WET model

A critical aspect of our research was the careful selection of animal models with suitable genetic backgrounds, immune statuses, and physiologies. To this end, we utilized BN and LEW rats to create the Syn and Allo WET models, respectively (**Figure 1**). The genetic consistency of these inbred strains provided a stable platform for our immunological investigations (38). Crucially, the significant polymorphic and polygenic variations within the major histocompatibility complex (MHC) between these strains are vital for eliciting distinct immune responses. The complete MHC mismatch between LEW and BN rats, in particular, offers an ideal scenario to study pronounced and clinically relevant rejection processes (39). Furthermore, we meticulously evaluated the restoration and ongoing maintenance of the ocular blood supply post-WET, using non-invasive methods such as monitoring limbal vessel reperfusion and employing Optical Coherence Tomography (OCT) to detect the central retinal artery and vein. Our observations confirmed that the transplanted eyes maintained a stable blood supply post-operatively, with IOP measurements post-transplantation remaining within the normal physiological range. This indicated successful restoration of blood volume, aqueous humor dynamics, and aqueous outflow. Additionally, using OCT and histological analysis, we confirmed the stable structural integrity of the transplants, further validating the success and suitability of our model.

### 4.2 Characterization of the WET immune response

#### 4.2.1 Early innate immune response

The early phase of the innate immune response in WET is characterized by a complex interplay of complement system activation, cytokine upregulation, and the infiltration of neutrophils and monocytes. This multifaceted response is evidenced by gene expression profiles, notably the upregulation of *C3* and *IL6*, and histological findings of polymorphonuclear and mononuclear MPO+ cells in ocular tissues. These elements collectively play a crucial role in the initial defense against transplant-related injury and in activating the adaptive immune response. The complement system, integral to the inflammatory processes associated with IRI, aids in T cell sensitization against donor antigens by donor antigen-presenting cells (APCs) and supports T cell proliferation, cytokine release, B cell maturation, and antibody production (40). It has been reported that MPO, an enzyme produced by neutrophils, some monocytes, and tissue macrophages, contributes significantly to early transplantation inflammation (41). Early inflammation in the conjunctiva consisted of both mononuclear and polymorphonuclear leukocytes, although only mononuclear cells were observed in the cornea at this phase. The notable increase in the expression of the proinflammatory cytokine *IL-6*, a key mediator of acute phase responses and inflammation, further underscores the activation of the innate immune environment and lays the groundwork for the activation of the adaptive immune response.

#### 4.2.2 Initiation of the adaptive response

By POD4, our histopathological analysis indicated an increase in CD3+ staining in the Allo transplanted eyes. This finding suggests the onset of a T cell-mediated immune response. Subsequent gene expression profiles on POD5 in the Allo group reinforced this observation, revealing a notable upregulation of the chemokine receptor *CXCR3* and its ligands *CXCL9, CXCL10*, and *CXCL11*. These chemokines play a pivotal role in the differentiation and migration of T helper 1 (Th1) cells, especially interferon-gamma (IFN-gamma)-secreting Th1 cells, to the graft site (42). The elevated levels of these chemokine genes, coupled with a concurrent increase in *IFN-gamma* (*IFNG*), highlight a significant Th1 cell-mediated rejection episode in WET. Additionally, the pronounced presence of cytotoxic T lymphocyte (CTL) markers such as *CTLA4* and *CD8A*, along with *TAP1* — integral for MHC class I antigen presentation and critical for presenting endogenous antigens to CTLs — emphasizes the vital role of CTLs in the rejection process in WET. The upregulation of genes directly related to cellular cytotoxicity, specifically *GZMA, GZMB*, and *PRF1*, suggests a primary involvement of CTLs in direct cell lysis. However, the contribution of Natural Killer (NK) cells to this increase in gene expression should also be considered.

#### 4.2.3 Early antibody-mediated immune response

In organ transplantation, the diagnosis of antibody-mediated rejection typically hinges on detecting DSAs and histopathological evidence of tissue injury (43). In our WET study, we noted the presence of serum IgM and IgG antibodies at specific post-operative time points. Coupled with the tissue damage observed in our histopathology analysis, these findings suggest a role for antibody-mediated rejection in the overall rejection process. The early emergence of these antibodies implies their contribution to the rapid rejection observed in WET, thus expanding our understanding of immunological responses in eye transplants. This insight is crucial for the development of targeted immunotherapies in transplantation. Moreover, the early detection of these antibodies serves as a vital diagnostic tool, helping identify rejection swiftly and allowing for prompt intervention. Timely and effective management of the immune response could significantly enhance outcomes in eye transplantation.

#### 4.2.4 Early evidence of cell death

Given the rapid graft rejection and shortened survival observed in this study, we specifically focused on genes associated with various cell death pathways. This emphasis was driven by histological evidence of cell death, as indicated by the necrosis observed in H&E staining. Notably, the marked upregulation of *CASP1*, a critical enzyme in pyroptosis, by 2.46-fold at POD2 and 23.67-fold at POD5, along with a significant rise in *IL1B*, suggests a potential role for pyroptosis in the graft rejection process. Pyroptosis is characterized by caspase-1 mediation and is distinct from apoptosis due to its rapid progression and intense inflammatory nature (44). It may be a key driver in the swift and severe immune-mediated damage leading to graft failure. This pathway has been implicated in the pathogenesis of transplant rejection in various organs, including kidneys, hearts, lungs, skin, and corneas (45, 46). While genes such as *CASP3*, *CASP8*, FAS, and *FASLG* were also upregulated, their fold changes were modest, ranging from 2.78 to 9.90. These comprehensive insights highlight the need for targeted strategies focusing on pyroptotic pathways. Addressing these mechanisms holds promise for developing more effective interventions to mitigate rapid graft rejection.

#### 4.2.5 Role of ischemia- reperfusion injury in allograft rejections

IRI is an inevitable component of organ transplantation. Its impact on transplantation outcomes is a significant area of research warranting dedicated exploration. In our study, we did not specifically target the acute aspects of IRI; however, we discovered indirect evidence suggesting its critical role in allograft rejection in eye transplantation. Initially, the increased opacity observed in some Syn group samples, devoid of histological signs of infiltration, prompted us to consider other contributing factors, such as IRI, which might alter corneal crystalline proteins and, consequently, impair transparency (47). The specific increase in serum IL-17A levels in both the Allo and Syn groups, frequently reported in IRI studies (48–51), further suggests a link with IRI post-WET. Moreover, the notably lower expression of the *ITGAE* (*CD103*) gene at POD2 and POD5 in the Allo group, and at POD2 in the Syn group, drew our attention. CD103 is expressed on dendritic cells (DCs) and corneal tissue-resident memory T cells (CD103+TRM) in ocular tissues. CD103+TRM is typically associated with prior inflammation-induced injury (52, 53), and CD103+ DCs are considered instrumental in the pathogenesis of IRI (54, 55). While DC depletion can have protective, harmful, or neutral effects as noted in a review (56), the observed decrease in *CD103* expression in our study might indicate a reduction in CD103+ DCs in the eye, potentially resulting from IRI-induced injury. However, the exact role of this alteration remains unclear and necessitates further investigation. This finding highlights the complexity of immune responses in WET and underscores the importance of continued research to fully understand the intricate mechanisms involved.

#### 4.2.6 Role of blood-retinal barrier

In discussing eye transplantation, a crucial consideration is the role of the BRB in rejection. The ocular microenvironment meticulously regulates the composition of its internal fluids and shields the inner ocular tissues from external disturbances via the blood-aqueous and blood-retinal barriers (57). The retina, protected under these mechanisms, is vital for ocular transplantation as its integrity directly influences visual outcomes. In our study, no immune privilege was observed; all allografts were rejected without exception. However, we noted that the BRB initially delayed immune-mediated retinal damage following WET, albeit temporarily. The choroid, unlike the retina, is not immune privileged. According to our study findings, the choroid is a likely starting point for rejection. Choroidal inflammatory mediators can disturb the BRB, allowing easier access for immune cells. Despite the BRB, there is always some minor cellular traffic in the retina, which can cascade once the barrier is perturbed. Eventually, immune cells penetrated this barrier, leading to retinal rejection, as evidenced by our histological analyses (**Figures 6I, J, L**). This penetration aligns with reports of irreversible retinal damage due to intraocular inflammation during graft rejection (58). Therefore, the period between the onset of graft rejection and the subsequent breach of the BRB is crucial for preserving retinal function. Our findings highlight the potential for therapeutic strategies aimed at reinforcing the protective role of the BRB. Enhancing its integrity could prolong the window before immune rejection affects the retina, offering a promising avenue for future research to optimize WET outcomes. Understanding and potentially augmenting the functions of the BRB could play a significant role in improving the prognosis for eye transplant recipients.

### 4.3 Diagnosis strategies for WET rejection

Effective diagnosis and monitoring of rejection are critical for the success of eye transplantation. Our study provides a detailed timeline of immune responses WET, emphasizing the early immune reactions and the distinct onset of rejection in both extraocular and intraocular tissues. For a comprehensive overview of the sequential immune responses and associated markers following WET, we have meticulously compiled a detailed timeline in **Table 1**. This table elucidates the dynamic immune processes occurring across various PODs and highlights potential biomarkers that are crucial for the early diagnosis and intervention in graft rejection. By integrating these diverse diagnostic approaches, we significantly enhance the accuracy and timeliness of rejection detection and monitoring in WET. Therefore, continuous monitoring post-transplantation is imperative for early intervention and reducing long-term adverse outcomes.

**Table 1:** Sequential immune responses and markers detection following WET.

	Tools/biomarkers	POD2	POD4	POD5	POD6	POD8
Type of immune responses determined by invasive tools	Ocular histopathology	Innate immune response	Innate/adaptive immune response	Innate/adaptive immune response	Innate/adaptive immune response	Innate/adaptive immune response
	Gene expression	Innate immune response		Innate/adaptive immune response		
Specific and non-specific changes determined by non-invasive tools/biomarkers	Serum DSAs		IgM increase	IgM Specific increase	IgG Specific increase	IgG Specific increase
	Serum CXCL10		Specific increase	Specific increase		
	Serum IFN- γ		Non-specific increase	Specific increase		
	Corneal transparency score	Non-specific increase	Non-specific increase	Specific increase	Specific increase	Specific increase
	Corneal thickness				Specific increase	Specific increase
	Skin rejection score		Non-specific increase	Specific increase	Specific increase	Specific increase

This table presents a timeline of immune responses and the detection of various markers following WET. It illustrates the dynamic immune processes over PODs and pinpoints potential biomarkers for early diagnosis and intervention in graft rejection. ‘Specific’ and ‘non-specific’ changes are defined based on a comparative analysis between the Allo group and the Syn control group, as discussed in the results section.

#### 4.3.1 Corneal transparency and vascular changes as monitoring indicators for WET

In human corneal transplantation, a standard scoring system from 0 to 4 is commonly used based on the whole corneal opacification to diagnose rejection (59). However, our WET model revealed non-uniform transparency loss, starting peripherally and progressing centrally. This peripheral initiation of opacity aligns with expectations, as the peripheral cornea contains blood vessels where rejection is likely to commence, contrasting with the avascular nature of typical human corneal transplants. In human cases, rejection primarily affects the stroma or endothelium initially, where opacity often results from diminished endothelial activity leading to intrastromal edema or fluid accumulation. Cellular infiltration, which causes opacity, is less common and typically manifests as a more uniform effect across the cornea. Consequently, to better represent the distinct pathophysiology observed in our animal model, we adapted the diagnostic criteria to account for this specific pattern of transparency loss (see **Figure 3**). From POD4, the Allo group demonstrated a notable rise in corneal opacity scores, consistent with pronounced histological evidence of immune cell infiltration (**Figure 7**). But as we have discussed before, due to the potential effect of IRI, the significant difference is delayed to POD5. Given the absence of immunosuppressive therapy in our study, both IRI and rejection might have concurrently influenced the cornea, affecting the reliability of transparency as a diagnostic marker. However, in settings with early immunosuppressive intervention, where rejection is typically suppressed or delayed, IRI effects would likely recede earlier, potentially making transparency a more specific indicator of rejection. Therefore, assessing corneal transparency changes holds promise as a diagnostic tool for WET, warranting further investigation.

The discussion on the utility of corneal thickness as a diagnostic measure for corneal transplant rejection is ongoing in both research and clinical settings (60, 61). In our study, significant changes in corneal thickness in WET animals were not observed until POD6. At this late stage, Allo group skin grafts displayed rejection grades of 3A with the presence of single-cell necrosis in the epidermis (**Figure 8**). This suggests that changes in corneal thickness are not an early sensitive indicator of rejection, exhibiting a significant delay.

Vascular endothelial cells, distinct from the corneal endothelium, are critical in organ transplantation, serving as the primary interface between the host’s immune system and the transplanted tissue. Often the initial targets during immune responses (62, 63), these cells reside in the strategically situated limbal vasculature, which bridges the inner and outer eye and enables effective immune surveillance. Our study detected early alterations in the limbal vasculature of both Syn and Allo groups by POD2. Notably, progressive vascular damage was exclusive to the Allo group, hinting at rejection. However, the variable temporal pattern of this damage among recipients complicates the use of these observations for consistent rejection grading. This variability underscores the need for more precise diagnostic techniques. Future research should focus on advanced imaging methods to provide a clearer and more quantifiable understanding of these vascular changes.

#### 4.3.2 Serum biomarkers as non-invasive diagnostic tools for WET rejection

In organ transplantation, the analysis of cytokines and chemokines within organ tissue, typically obtained through biopsies or by analyzing tissue-specific secretions, plays a significant role in predicting or diagnosing rejection (64–67). However, these methods are not feasible in the context of WET due to the unique anatomical and physiological characteristics of the eye, necessitating the exploration of alternative diagnostic approaches for detecting and monitoring rejection in WET. Our study focused on investigating serum cytokine/chemokine levels in WET, particularly their roles in the rejection process. A key finding in our study was the marked and specific elevation in CXCL10 levels at the early stages post-Allo WET. Also known as Interferon Gamma-Induced Protein 10 (IP-10), CXCL10 plays a crucial role in promoting T cell migration and activation. This likely reflects the intensified immune activity within the transplanted organ (68). The observed increase in serum CXCL10 coincided with a significant up expression of *CXCL10* genes and increased T cell infiltration in the eye. This suggests a direct correlation with an active immune mechanism, underscoring the potential of CXCL10 as a predictive and diagnostic biomarker for WET rejection. This aligns with findings from other organ transplantation research, where CXCL10 has been recognized as an important biomarker with both diagnostic and predictive value for early rejection (69–71). Conversely, the early non-specific increase in IFN-γ levels on POD4 in the Allo group, compared to the Syn group, could be attributed to a combination of IRI and rejection. While the sustained elevation of serum IFN-γ at POD5 may indicate WET rejection, current studies suggest that changes in IFN-γ levels are not consistently correlated with rejection and can be influenced by other factors such as infection and inflammation (72, 73). Although there was a general increase in other cytokines/chemokines in both groups, likely reflecting the combined effects of IRI and the transplant immune response, sufficient evidence to support their use as noninvasive diagnostic markers for predicting rejection in WET was not found (74–77). This study underscores the potential of serum CXCL10 as a non-invasive biomarker for early detection and monitoring of rejection in WET, contributing to the advancement of diagnostic strategies in ocular transplantation.

### 4.4 Study limitations and future directions

Recognizing the limitations of this study is crucial for accurately interpreting the findings and guiding future research directions. A primary limitation is the lack of an established baseline for immunosuppressive treatment in Whole Eye Transplantation (WET), which hinders the ability to directly compare these results with established transplantation models. Additionally, the small sample sizes, necessitated by the complexity of the WET model, may limit the generalizability of our findings. Furthermore, this study does not delve into the most detailed molecular mechanisms or cover every aspect of immune cell interaction and signaling pathways, areas which hold potential for future exploration.

Despite these limitations, the research still provides significant insights into the temporal and functional changes in the immune system post-transplantation, which are central to understanding immune dynamics in WET. Future research should focus on exploring diverse immunosuppressive and immune tolerance induction strategies specifically tailored to counteract transplant rejection while protecting the retina and the BRB. The potential of IL-6 as an immune-modulatory target (78), alongside the development of complement-targeted treatments in WET, presents promising avenues for novel immunotherapy approaches in eye transplantation. Further investigation into the role of IRI in allograft rejection is also identified as a crucial area, which could significantly enhance graft survival and improve transplantation outcomes. Additionally, the corneal transparency grading system developed in this study can be further validated by systematically correlating biomarkers, such as serum CXCL10, with specific transparency scores, thereby establishing a quantitative link between biological markers of rejection and clinical observation. Delving into detailed molecular mechanisms and comprehensive coverage of immune cell interactions and signaling pathways in future studies will also be vital to fully understand and address the complexities of immune responses in WET.

## 5 Conclusions

This comprehensive study on WET has unveiled vital insights into the immune mechanisms of graft rejection and established pioneering diagnostic and monitoring strategies. Through the detailed characterization of immune responses in both Syn and Allo WET models, we have highlighted the rapid and dynamic nature of these responses. Our findings demonstrate an early innate immune response, robust T cell-mediated reactions, and involvement of antibody-mediated mechanisms in the rejection process. The study also underscores the importance of the unique ocular environment, especially the role of the BRB in delaying immune-mediated retinal damage. Novel diagnostic criteria, based on corneal changes and serum biomarker analysis, particularly the potential of CXCL10 as an early biomarker for rejection, represent significant advancements. This research provides a foundational understanding of the immune dynamics in WET and sets the stage for future explorations into immunosuppressive strategies, crucial for enhancing graft survival and optimizing outcomes in eye transplantation. However, further studies are necessary to evaluate visual function, optic nerve outcomes, and the impact of immunosuppressive therapies to better translate these findings into clinical relevance for human transplantation.

## Data Availability

The original contributions presented in the study are included in the article/supplementary material, further inquiries can be directed to the corresponding author/s.
