# Supplementary material for: Immune responses in rodent whole eye transplantation: elucidation and preliminary investigations into rejection diagnosis and monitoring
Source: Front Immunol. 2025 Jan 29;16:1475055. doi: 10.3389/fimmu.2025.1475055 (PMC11814173; doi:10.3389/fimmu.2025.1475055)
Supplement: Supplementary file 1 [file DataSheet1.pdf]

# *Supplementary Material*

## Supplementary Table

| Timepoints / Groups | | POD0 | POD2 | POD4 | POD5 | POD6 | POD8 | Total |
|---|---|---|---|---|---|---|---|---|
| **Cross-sectional** | Allo | - | 9* | 6 | 9* | 6 | 6 | 36 |
| | Syn | - | 6* | 3 | 6* | 3 | 3 | 21 |
| | Naïve | 9* | - | - | - | - | - | 9 |
| **longitudinal** | Allo | 5 | 5 | 5 | 5 | 5 | 5 | 5 |
| | Syn | 5 | 5 | 5 | 5 | 5 | 5 | 5** |

**Supplementary Table 1**. Experimental groups and animal allocation in the study. The cross-sectional group comprised 36 Allo and 21 Syn animals. For histological analysis, 6 Allo and 3 Syn animals were used at each timepoint, complemented by 6 naïve animals as controls. For quantitative PCR (qPCR) analysis, 3 Allo and 3 Syn animals were utilized on POD2 and POD5, along with 3 naïve animals serving as controls. The longitudinal study involved 5 Allo and 5 Syn animals, monitored over an 8-day period with examinations and sample collections at timepoints 0, 2, 4, 5, 6, and 8 days post-operation, as applicable.

* qPCR studies were conducted on 3 animals from selected timepoints in each group.

** After collecting all the designated data and samples, 3 of the 5 Syn animals were kept for 30 days to evaluate the restoration and long-term stability of blood supply, IOP, and structural integrity in the WET rodent model.

## Supplementary Figure

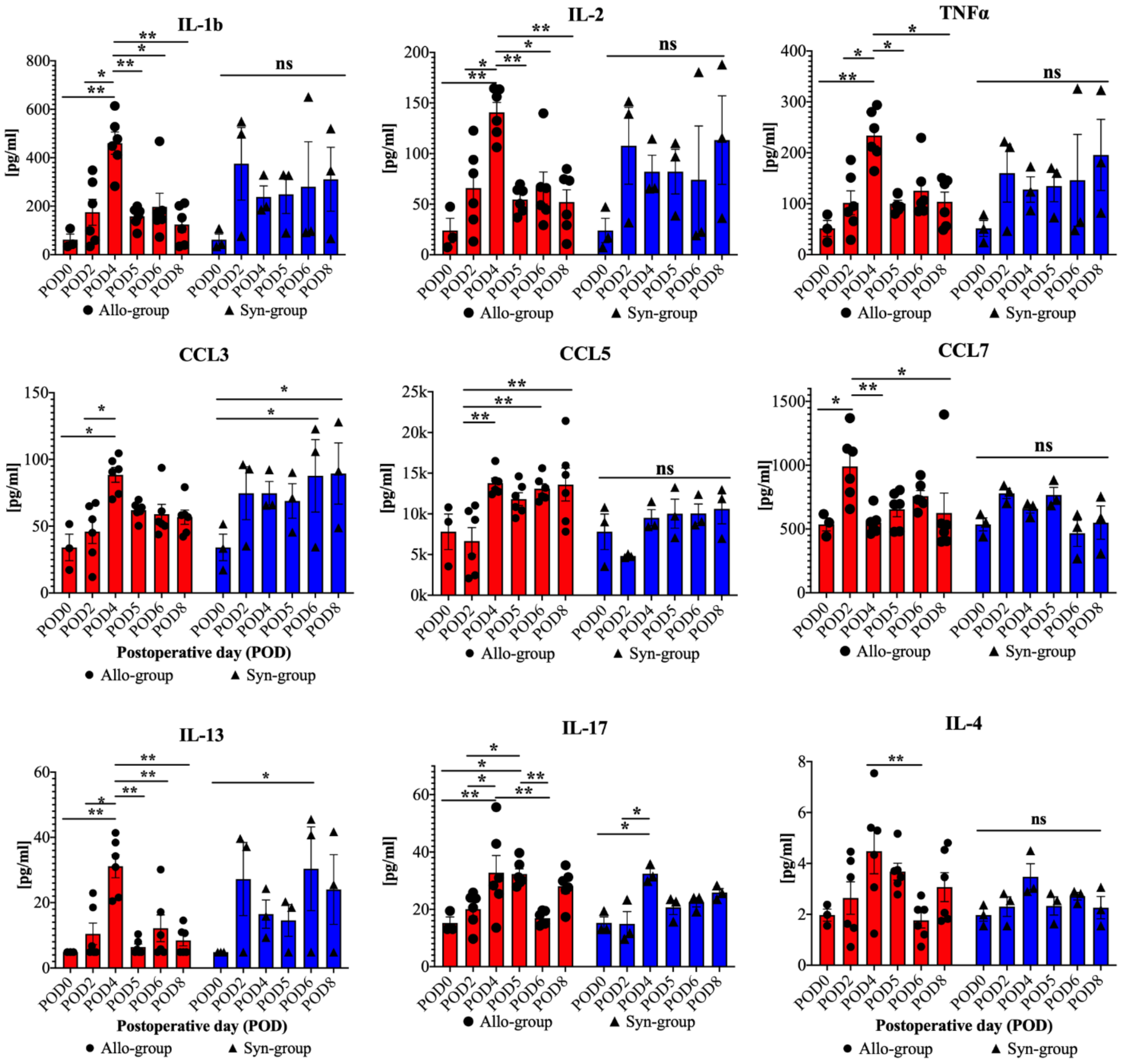


**Supplementary Figure 1.** Serum cytokine and chemokine levels post-Whole Eye Transplantation (WET). Serum levels of IL-1β, IL-2, TNF-α, CCL3, CCL5, CCL7, IL-13, IL-17, and IL-4 are shown for both allogeneic (Allo) and syngeneic (Syn) groups across various postoperative days (POD). Notable increases were observed in the Allo group, with specific rises in CCL3, IL-13, and IL-17 levels also noted in the Syn group (n=6/time-point for Allo, n=3/time-point for Syn). Groups were compared using two-way ANOVA, followed by Tukey's post hoc tests for multiple comparisons. Significance levels are denoted as ns (not significant), *P<0.05, **P<0.01.
